# The impact of the COVID-19 pandemic on trends in stillbirths, under-5 and maternal mortality in Brazil: Excess deaths and regional inequalities

**DOI:** 10.7189/jogh.13.06040

**Published:** 2023-09-30

**Authors:** Mariana Otero Xavier, Agbessi Amouzou, Abdoulaye Maïga, Nadia Akseer, Luis Huicho, Alicia Matijasevich

**Affiliations:** 1 Departamento de Medicina Preventiva, Faculdade de Medicina FMUSP, Universidade de São Paulo, São Paulo, Brasil; 2 Department of International Health, Johns Hopkins University Bloomberg School of Public Health, Baltimore, Maryland, USA; 3 Centro de Investigación en Salud Materna e Infantil, Centro de Investigación para el Desarrollo Integral y Sostenible and Facultad de Medicina, Universidad Peruana Cayetano Heredia, Lima, Peru

## Abstract

**Background:**

Despite the proliferation of studies on the impact of the coronavirus disease 2019 (COVID-19) pandemic, there is less evidence on the indirect death toll compared to the health system and service provision disruptions. We assessed the impact of the COVID-19 pandemic on national and regional trends and differences in stillbirths, under-5 and maternal deaths in Brazil.

**Methods:**

We used the nationwide routine health information system data from January 2017 to December 2021, to which we applied descriptive and advanced mixed effects ordinary least squared regression models to measure the percent change in mortality levels during the COVID-19 pandemic (March 2020 to December 2021). We carried out counterfactual analyses comparing the observed and expected mortality levels for each type of mortality at national and regional levels.

**Results:**

Stillbirths increased 4.8% (3.1% in 2020 and 6.2% in 2021) and most noticeably maternal deaths increased 71.6% (35.3% in 2020 and 103.3% in 2021) over the COVID-19 period. An opposite pattern was observed in under-5 mortality, which dropped -10.2% (-12.5% in 2020 and -8.1% in 2021). We identified regional disparities, with a higher percent increase in stillbirths observed in the Central-West region and in maternal deaths in the South region.

**Discussion:**

Based on pre-pandemic trends and expected number of deaths in the absence of the COVID-19, we observed increases in stillbirths and maternal deaths and reductions in under-5 deaths during the pandemic. The months with the highest number of deaths (stillbirths and maternal deaths) coincided with the months with the highest mortality from COVID-19. The increase in deaths may also have resulted from indirect effects of the pandemic, such as unavailability of health services or even reluctance to go to the hospital when necessary due to fear of contagion.

**Conclusions:**

In Brazil, the COVID-19 outbreak and subsequent restrictions had a detrimental impact on stillbirths and maternal deaths. Even before the pandemic, mortality trends highlighted pre-existing regional inequalities in the country's health care system. Although there were some variations, increases were observed in all regions, indicating potential weaknesses in the health system and inadequate management during the pandemic, particularly concerning pregnant and postpartum women.

The coronavirus disease 2019 (COVID-19) pandemic has had direct and indirect impacts on population health and threatened countries’ progress in achieving the Sustainable Development Goals (SDGs) [1]. From the onset of the pandemic, we observed many studies on the impact of COVID-19 on service disruptions with a focus on the health system and service provision [2,3]. The death toll represents a critical indicator for assessing the impact of the pandemic, although the lack of reliable data at country level hampers studies focused on the analysis of mortality.

By December 2021, two years after the onset of the pandemic, reported COVID-19 deaths totalled 5.94 million worldwide [4]. Estimates of excess mortality, that is the number of deaths above the expected value following a previously observed mortality pattern, suggested that the impact of COVID-19 had been even more devastating, with about 18 million excess deaths between January 2020 and December 2021 [5]. The first case of COVID-19 in Brazil was recorded on 25 February 2020 and the death toll was an estimated 620 000 deaths by December 2021 [4].

Stillbirths, under-5 and maternal mortality are population-level indicators during antenatal and perinatal periods and in the early years of a child's life, and they are strongly associated with access to health services and socioeconomic inequalities. The stillbirth rate has been slowly falling worldwide [6], including in Brazil [7]. Similarly, the global under-5 mortality rate (U5MR) declined by 59% from 1990 to 2021 [8] and the maternal mortality ratio (MMR) dropped by about 38% worldwide between 2000 and 2017 [1]. Under-5 and maternal mortality also decreased over the years before the pandemic in Brazil, with reductions in regional disparities [9,10].

Despite substantial progress on reducing maternal and child mortality worldwide, the Millennium Development Goals 4 and 5 (a two third reduction in U5MR and a 75% reduction in MMR by 2015, respectively) were not achieved in most countries, among them Brazil that reached the goal only for U5MR [9,10]. Thus, global reductions of U5MR to less than 25 per 1000 live births and MMR to less than 70 deaths per 100 000 live births are two priority goals of the 2030 Agenda for SDGs [1]. Brazil established adjusted targets for 2030 to reduce U5MR to eight deaths by 1000 live births and MMR to 30 by 100 000 live births [11]. These goals require considerable efforts in the coming years, especially because of the emergence of the pandemic. Systematic reviews and multicentric studies showed that global maternal, foetal and child outcomes have worsened during the COVID-19 pandemic, including increasing stillbirths, neonatal and maternal deaths, among other adverse outcomes [12,13]. Some studies showed higher risks of maternal morbidity and mortality associated with COVID-19 [14-16]. However, the relationship is still unclear regarding stillbirths and under-5 mortality. Moreover, within the context of Brazil, there is limited evidence to-date on the impact COVID-19 has had on health service utilisation and health and survival outcomes.

The objective of this study was to assess the impact of the COVID-19 pandemic on national and regional trends and differences in stillbirths, under-5 and maternal deaths in Brazil using routine health information system data from January 2017 to December 2021. The estimated monthly changes in mortality were also correlated with the monthly number of COVID-19 deaths officially reported and to the government response stringency index of COVID-19 restriction measures. Lastly, we analysed changes in U5MR and MMR based on a historical time-series from 2010 to 2021. Results from this analysis provide critical insights into the magnitude of COVID-19’s impact on women’s and children’s survival, which would be valuable for policymakers and health professionals in practice and for future pandemic preparedness.

## METHODS

### Study design and data sources

We applied an ecological design to study the key research questions. We used the Mortality Information System data from the National Health System Information Technology Department (DATASUS) of the Ministry of Health of Brazil, which provided absolute counts of stillbirths, under-5 and maternal deaths [17]. Brazil is the largest country in South America and the fifth most populous in the world, with an estimated population of about 213 million inhabitants in 2021. The country is composed of 27 federation units, divided into five geographic regions: North, Northeast, Central-West, Southeast, and South [18].

The total number of deaths due to COVID-19 and the stringency index were extracted from Our World in Data [4]. The stringency index measures the strictness associated with containment restrictions and public information campaigns across countries and over time. It is a composite measure based on nine response metrics including school closures, workplace closures and travel bans, cancellation of public events, restrictions on public gatherings, closures of public transport, stay-at-home requirements, public information campaigns and restrictions on internal movements, rescaled to a value from 0 to 100 (100 = strictest response) [19]. The estimated population by year and by federation unit was obtained from projections made by the Brazilian Institute of Geography and Statistics (IBGE in Portuguese) [18]. The period of extraction and organisation of data in spreadsheets and databases for analysis occurred between June and September 2022.

### Operational definitions

We analysed three key outcomes in this study, including stillbirths, under-5 child death and maternal death. These indicators were defined using standardised definitions. According to the criteria adopted by the Ministry of Health of Brazil, stillbirth is the death or loss of the foetus before or during delivery that has reached a birth weight of 500 grammes (g), or if birth weight is unavailable, gestational age of 22 weeks (154 days) or more. When information on birth weight and a gestational age is not available, crown-to-heel length of 25 cm or more should be considered. The foetus is presumed dead if it shows no breathing signs or any other vital sign, such as beating of the heart, umbilical cord pulsations or effective movements of voluntary muscles after delivery [20]. Under-5 death is the death of a child before reaching their 5th birthday.

Maternal death is defined as the death of a woman which occurs during pregnancy or within 42 days after the end of the pregnancy, regardless of pregnancy duration or location, due to any cause related to or aggravated by pregnancy itself or situations regarding it, but not due to accidental or incidental causes [21]. We extracted monthly data on stillbirths, under-5 deaths and maternal deaths on a monthly basis from January 2017 to December 2021. Numbers from 2021 are preliminary and could still be updated.

The MMRs, defined as the number of maternal deaths per 100 000 live births of mothers residing in a given geographic area in the considered year, were extracted from the mortality indicator panel from the Ministry of Health of Brazil [22] from the year 2010 to 2021. Up to 2020 the MMRs were calculated by the Ministry of Health using correction factors, considering the existence of underreporting and failures in the process of investigation of maternal deaths [22]. As data from 2021 are still preliminary, the MMR was directly calculated using the available numbers in DATASUS of maternal deaths and live births for 2021, with no correction factors.

The U5MRs were also calculated and extracted from the Ministry of Health of Brazil using the number of deaths of children under five years residing in a given geographic area in the considered year (from the year 2010 to 2021), divided by the number of live births of resident mothers, multiplied per 1000 [22]. From 2014 to 2020 the U5MRs were calculated using correction factors for underreporting. However, between 2010 and 2013 and for 2021 data presented were directly calculated without using correction factors [22].

### Statistical analysis

Initially, we conducted a descriptive analysis, presenting the medians and interquartile ranges of the monthly number of deaths for the periods before and during the pandemic. Correlation analyses were then applied to assess the relationship between each type of death (stillbirths, under-5 and maternal deaths) and the number of deaths from COVID-19 in Brazil from March 2020 to December 2021.

We calculated and compared the monthly number of each type of death: stillbirths, under-5, and maternal deaths. The trend and counterfactual analyses consisted of comparing the pre-pandemic period (January 2017 to February 2020) with the pandemic period (March 2020 to December 2021). This involved presenting graphic representations of time series and displaying the observed and expected number of deaths with 95% confidence intervals (95% CI).

We used mixed effects ordinary least squared regression models to estimate the size of any change in deaths for each COVID-19-month from March 2020 to December 2021 and for the whole COVID-19 period in 2020-2021. First, we fitted a model to the entire period (January 2017 to December 2021) to establish the observed trends. Next, we fitted the same model to the pre-COVID-19 period (January 2017 to February 2020). Using this second model, we predicted the number of deaths for each outcome in the absence of the COVID-19 expected deaths. We fitted these regressions using the monthly number of each type of death (stillbirths, under-5 and maternal deaths) at national and regional levels as the dependent variable (*Y_ij_*). We used federation units as units of analysis. This dependent variable (*Y_ij_*) was regressed on a time variable (time) defined in months (from January 2017 to December 2021) to capture time trends, each calendar month (Month) was included in the model as a dummy variable to control for seasonality in mortality, federation unit population (Pop), first administrative level (region) and COVID-19-month from March 2020 to December 2021 (covid month). The mixed effects models included random intercept and slope for time trends, accounting for multiple measurements at the federation unit levels.


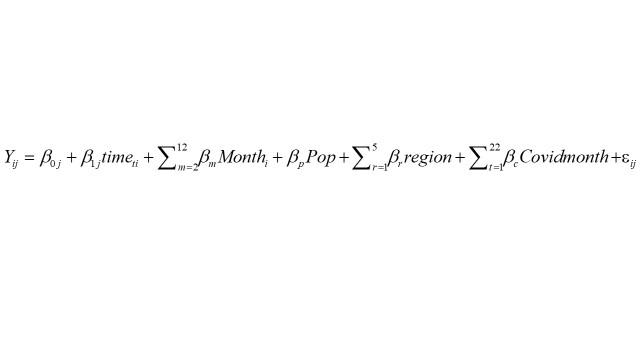
.(1)

The percent change values for each month/y of the pandemic were obtained using the equation: (100*(observed number of deaths – expected number of deaths)/expected number of deaths)). We computed the percent change in each type of death during COVID-19 months at national and regional levels.

The predicted (expected) values of MMR and U5MR for the pandemic years (2020 and 2021) were estimated from segmented linear regressions, including the pre-pandemic period (2010 to 2019) as exposure and MMR and U5MR as outcomes. The models were fitted using STATA 16.0 software (StataCorp., College Station, TX, USA). Results are considered significant at the *P* < 0.05 level for two-sided comparisons.

### Ethics

In accordance with article 1 of resolution 510 / 2016 of the National Research Ethics Commission, the study did not require approval from the Research Ethics Committee for using exclusively secondary data of public access, and without the possibility of identifying individuals.

## RESULTS

### Descriptive findings

**Table 1** and **Table 2** show a brief description of the data. There were some declines in the median monthly number of stillbirths (2505 to 2400) and under-5 deaths (3435 to 2989) comparing the pre-pandemic and pandemic periods. However, the median monthly number of maternal deaths increased from 137 to 173 over these periods (**Table 1**). A total of 22 291 839 cases of COVID-19 and 619 334 deaths from COVID-19 were reported between February 2020 and December 2021 in Brazil. The overall average of the stringency index over this period was 61.0% (standard deviation (SD) = 15.8) (**Table 2**).

**Table 1:** Descriptive statistics in the pre-pandemic (January 2017 to February 2020) and during the pandemic (March 2020 to December 2021) periods in Brazil

	Pre-pandemic (n = 38 months)	During the pandemic (n = 22 months)	Ratio (pandemic/pre-pandemic)
	**Median monthly number (IQR)**	**Median monthly number (IQR)**	
Stillbirth	2505.0 (233.0)	2399.5 (216.0)	0.96
Under-5 deaths	3434.5 (345.0)	2989.0 (179.0)	0.87
Maternal deaths	137.0 (18.0)	172.5 (102.0)	1.26

IQR – interquartile range (Q75-Q25)

**Table 2:** Characteristics of the country, cumulative numbers of COVID-19 cases and deaths, and mean containment stringency index

	Number of subnational units used for analysis (federation units)	Estimated population in 2021*	Cumulative number of reported COVID-19 cases (Feb-2020 to Dec-2021)†	Cumulative number of reported COVID-19 deaths (Feb-2020 to Dec-2021)†	Mean (SD) containment stringency index (Feb-2020 to Dec-2021)†
**Brazil**	27	213 317 639	22 291 839	619 334	61.0 (15.8)
North	7	18 906 962	1 923 966	47 550	
Northeast	9	57 667 842	4 950 128	120 018	
Southeast	4	89 632 912	8 662 789	294 659	
South	3	30 402 587	4 348 954	97 519	
Central-West	4	16 707 336	2 406 002	59 588	

SD – standard deviation

*Brazilian Institute of Geography and Statistics (IBGE) – Projection of the population of Brazil [22].

†Our World in Data [4].

**Figure 1:**
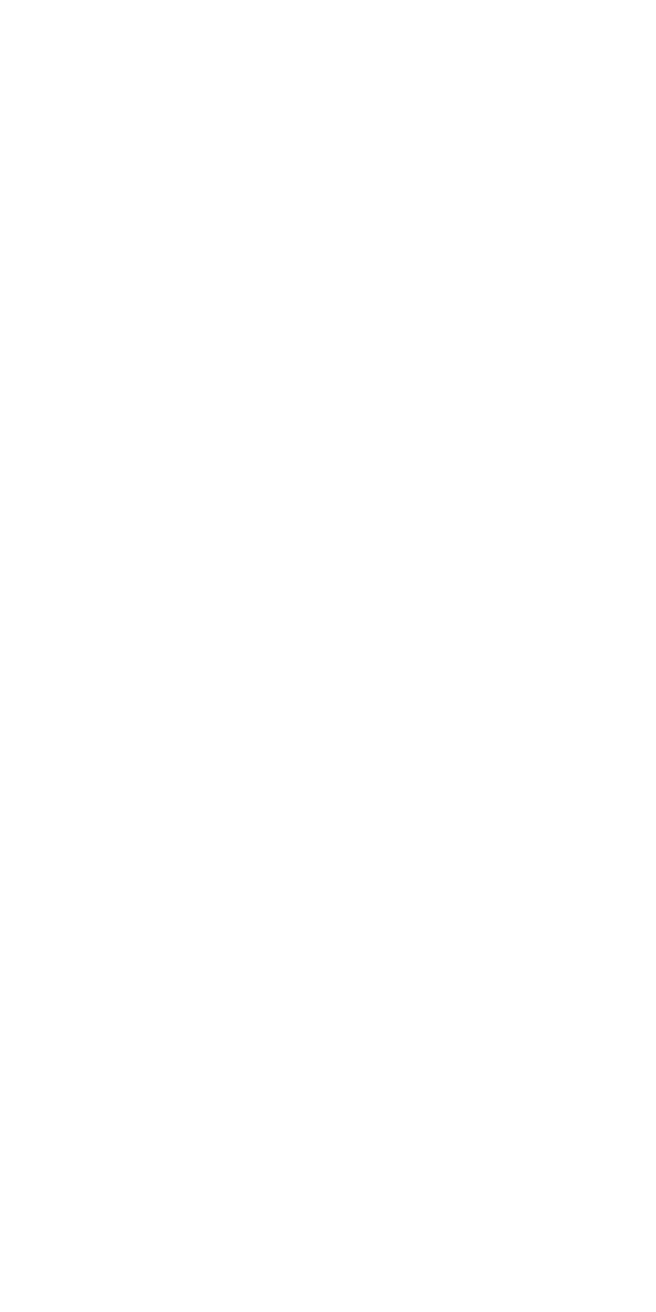
Total stillbirths, under-5 deaths and maternal deaths by month, January 2017 to December 2021 and COVID-19 deaths by month, February 2020 to December 2021 in Brazil. **Panel A.** Stillbirths. **Panel B.** Under-5 deaths. **Panel C.** Maternal deaths. Red dashed line represents the beginning of the COVID-19 pandemic.

**Figure 1** shows the total stillbirths, under-5 deaths and maternal deaths by month from January 2017 to December 2021 and COVID-19 deaths between February 2020 and December 2021. The second peak of COVID-19 deaths coincides with a slight increase of stillbirths between March 2021 and April 2021 (**Figure 1**, panel A). No apparent increase was observed in under-5 deaths during the pandemic period (**Figure 1**, panel B). Increases in maternal deaths clearly followed the two highest peaks of COVID-19 deaths (**Figure 1**, panel C). Correlation analyses revealed that maternal deaths were strongly and significantly correlated with deaths from COVID-19 (pearson's correlation coefficient (r) = 0.93; *P* < 0.001), while stillbirths showed a moderate correlation (r = 0.51; *P* = 0.012), and under-5 deaths exhibited a weak and non-significant correlation with deaths from COVID-19 (r = -0.02; *P* = 0.92).

**Figure 2:**
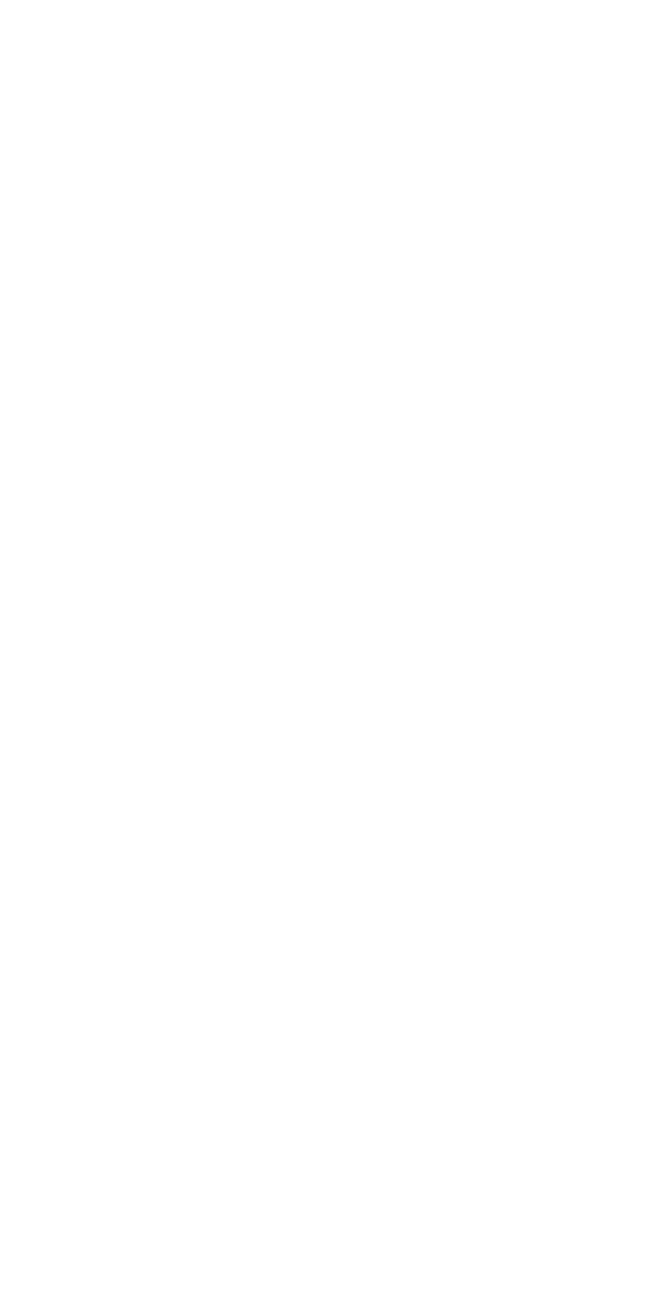
Observed and expected stillbirths, under-5 and maternal deaths from January 2017 to December 2021 in Brazil. **Panel A.** Stillbirths. **Panel B.** Under-5 deaths. **Panel C.** Maternal deaths. Red line represents the beginning of the COVID-19 pandemic.

The expected numbers for stillbirths were lower than the observed numbers from March 2020 to December 2021, while we observed a reverse pattern for under-5 deaths (**Figure 2**, panel A and panel B). The difference was much higher for maternal deaths with more observed deaths than expected during the pandemic (**Figure 2**, panel C).

### COVID-19 stringency index and monthly percent changes in death data

**Figure 3:**
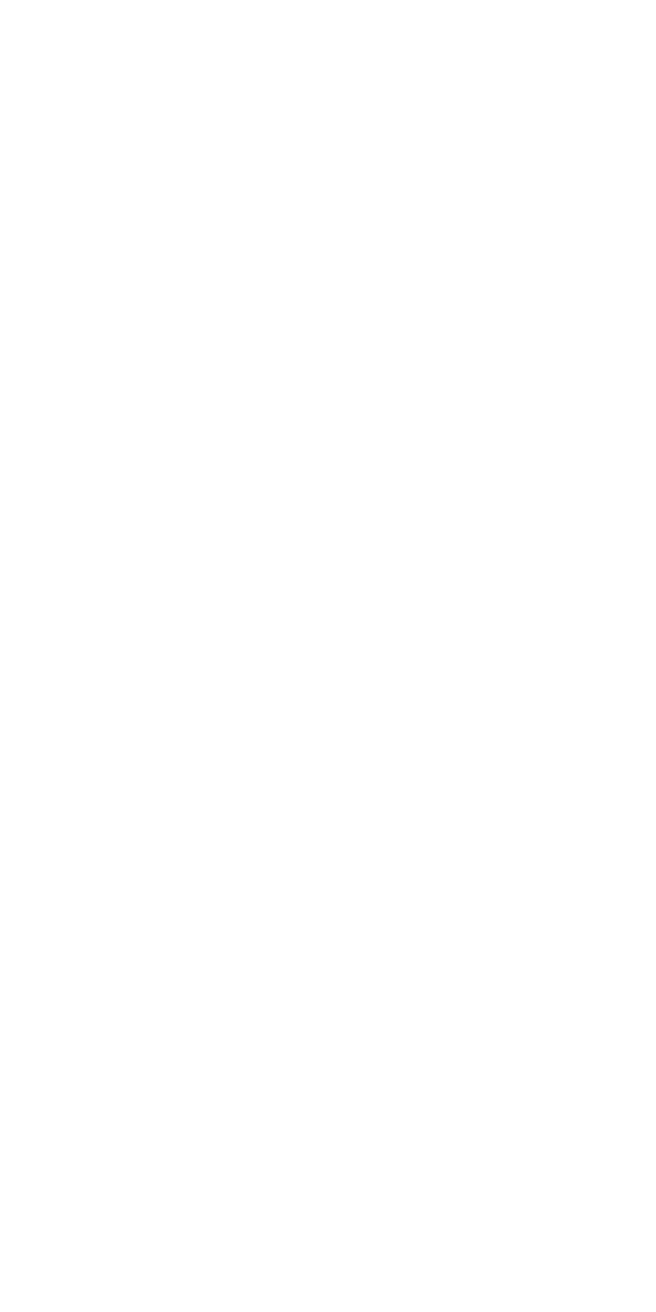
Mean stringency index for COVID-19 restrictions by month and percent changes (%) in stillbirths, under-5 and maternal deaths from March 2020 to December 2021 in Brazil compared to the expected deaths based on the preceding three years. **Panel A.** Stillbirths. **Panel B.** Under-5 deaths. **Panel C.** Maternal deaths. Higher stringency index indicates stronger restrictions.

Positive percentages suggest an increasing number of deaths while negative percentages indicate a decline. We observed more stillbirths than expected between April 2020 and December 2021, with the exception of October 2020 and November 2020. In general, percent changes in stillbirths were positively associated with the level of the stringency index (**Figure 3**, panel A). The number of under-5 deaths dropped over the study period (fewer deaths than expected). The largest drops occurred between June 2020 and July 2020 (when the stringency index was at its highest level) and between April 2021 and May 2021 (when the stringency index was decreasing) (**Figure 3**, panel B).

Observed maternal deaths were higher than expected (**Figure 3**, panel C). The highest percent increases occurred during the months of March 2021 and June 2021. Considering the whole COVID-19 period (March 2020 to December 2021) compared to the pre-pandemic period (January 2017 to February 2020), the increase in stillbirths and maternal deaths was 4.8% (3.1% in 2020 and 6.2% in 2021) and 71.6% (35.3% in 2020 and 103.3% in 2021), respectively, while under-5 deaths showed a reduction of -10.2% (-12.5% in 2020 and -8.1% in 2021) (Table S1 in the **Online Supplementary Document**).

**Figure 4:**
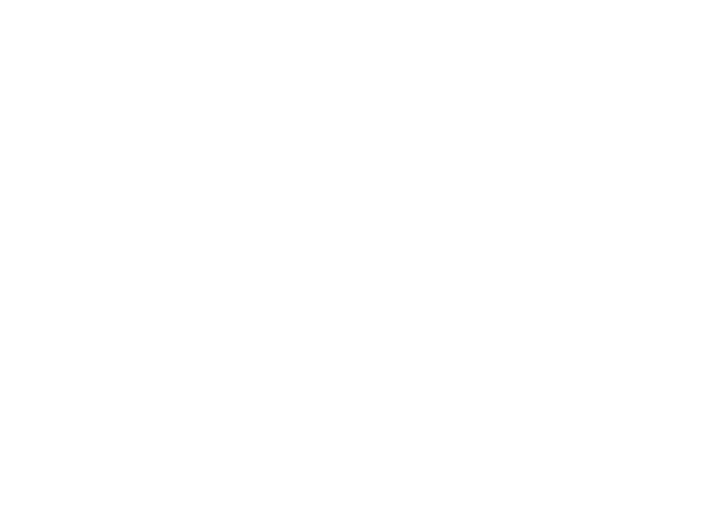
Percent change in stillbirths, under-5 deaths and maternal deaths from March 2020 to December 2021 by region compared to the expected deaths based on the preceding three years in Brazil. **Panel A.** Stillbirths. **Panel B.** Under-5 deaths. **Panel C.** Maternal deaths.

We also calculated the percent changes in stillbirths, under-5 deaths and maternal deaths by month and by region during the COVID-19 period (**Figure 4** and Table S2 in the **Online Supplementary Document**). Considering the total COVID-19 period, the highest percent increase for stillbirths was observed in the Central-West region (percent change of 11.6%; 8.7% in 2020 and 14.0% in 2021). The regions that showed the highest percentages of reductions in under-5 deaths were the Southeast (percent change of -13.2%; -14.9% in 2020 and -11.8% in 2021) and South (percent change of -11.8%; -15.0% in 2020 and -9.2% in 2021). Maternal deaths increased most in the South region (percent change of 111.1%; 25.8% in 2020 and 183.1% in 2021) (Figure 4 and Table S2 in the **Online Supplementary Document**).

### MMR and U5MR

**Figure 5:**
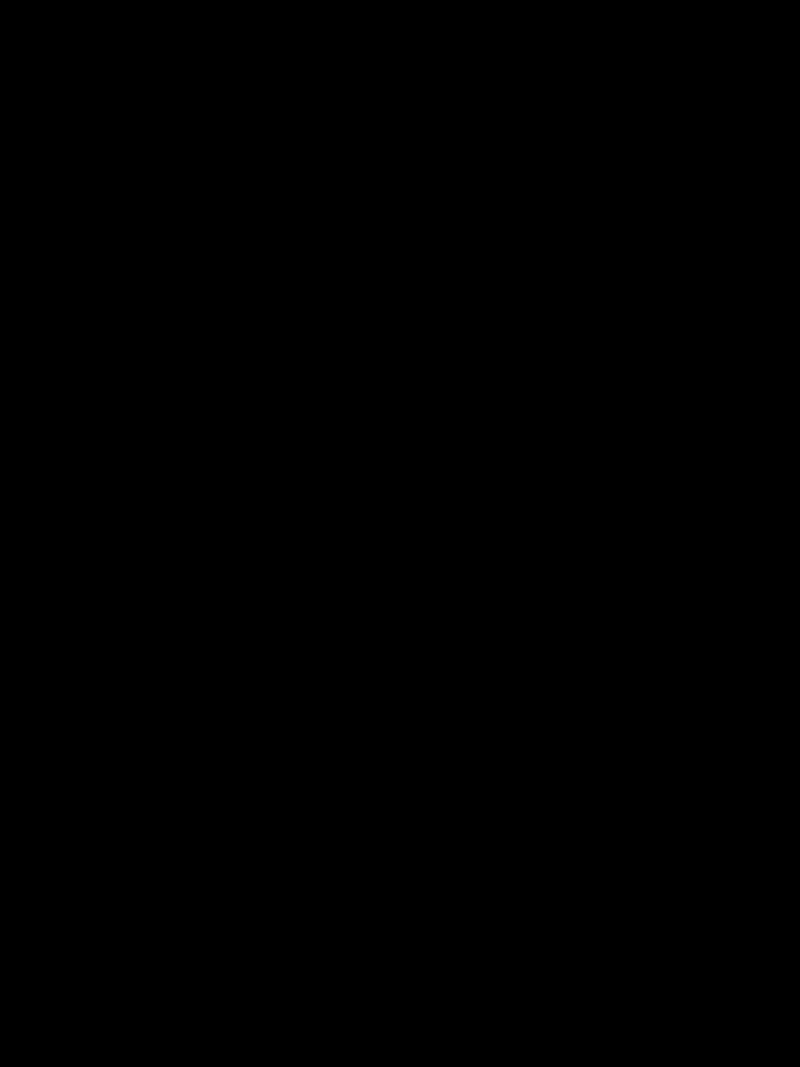
Maternal mortality ratio (MMR) in Brazil between 2010 and 2021. The dashed line delimits the last value (2019) before the start of the COVID-19 pandemic. The red line indicates the expected MMR values based on the previous years (2010-2019). *2021 MMR was calculated with no correction factors for underreporting.

**Figure 6:**
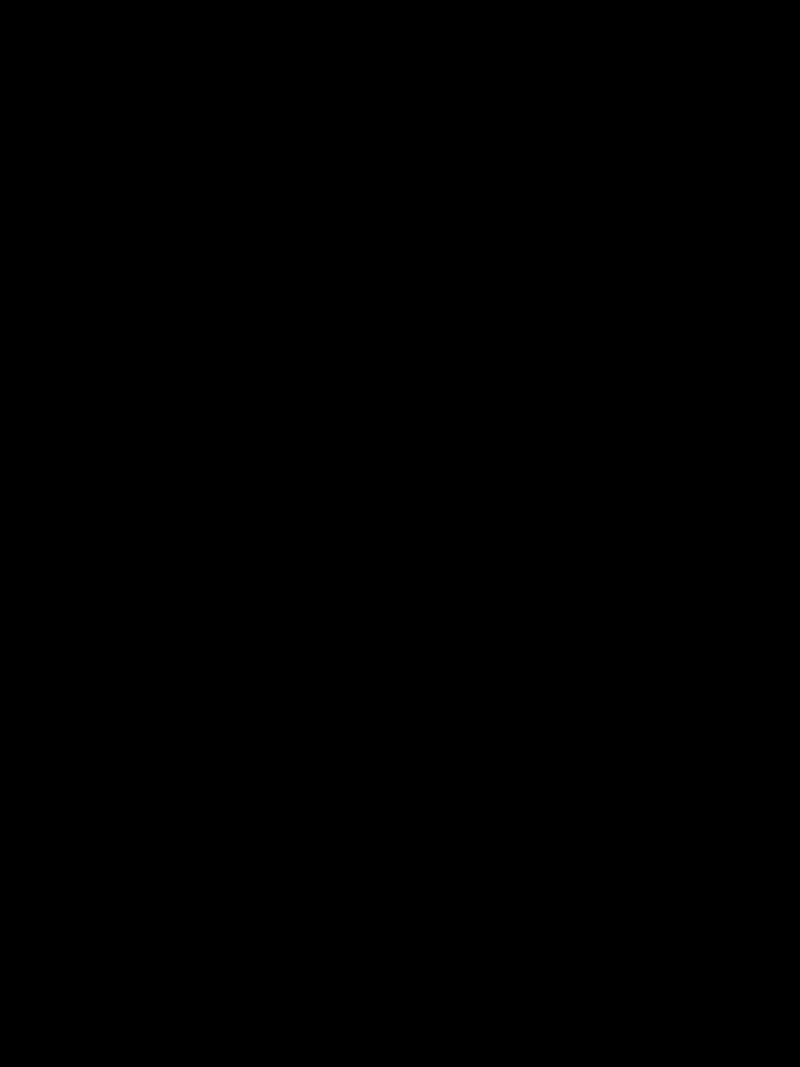
Under-5 mortality rate (U5MR) in Brazil between 2010 and 2021. The dashed line delimits the last value (2019) before the start of the COVID-19 pandemic. The red line indicates the expected U5MR values based on the previous years (2010-2019). *2021 U5MR was calculated with no correction factors for underreporting.

**Figure 5** shows the MMR in Brazil between 2010 and 2021. In the years prior to the pandemic, the MMR was decreasing in most regions, but there were notable increases during the pandemic, particularly in 2021, when the highest value was observed in the North region (MMR = 140.9) and the lowest in the Southeast region (MMR = 100.5). If the previous trends were maintained, the expected values of MMR in 2021 in the North and in the Southeast regions would be 87.3 (95% CI = 76.0-98.5) and 57.6 (95% CI = 50.1-65.1), respectively (**Figure 5** and Table S3 in the **Online Supplementary Document**). U5MR, which had also been showing reductions in the years prior to the pandemic in most regions of Brazil, followed a similar or even greater reduction trend after the beginning of the pandemic (**Figure 6**). The lowest U5MR was observed in 2021 in the South region (U5MR = 11.0) and the highest in the North region (U5MR = 17.5). In the hypothetical scenario of the same trend as the previous years, the U5MR values in 2021 in the South and North regions would be 10.9 (95% CI = 10.5-11.4) and 18.4 (95% CI = 17.1-19.6), respectively (**Figure 6** and Table S3 in the **Online Supplementary Document**).

## DISCUSSION

Our analyses showed trends of reduction in stillbirths and maternal deaths in Brazil during the pre-pandemic period. With the emergence of the pandemic, these trends were reverted, especially for maternal deaths. Compared to the expected number of stillbirths, under-5 and maternal deaths based on pre-pandemic trends, we observed increases in stillbirths and maternal deaths and reductions in under-5 deaths. The magnitudes of the percent changes varied by regions, with highest observed increases in stillbirths and maternal deaths in the Central-west and South regions, respectively. The months with the highest number of deaths (stillbirths and maternal deaths) coincided with the months with the highest mortality from COVID-19.

The Sistema Único de Saúde (SUS), Brazil's national health system, was implemented in 1990 and comprises a variety of public and private organisations with the goal of ensuring universality, equity, and quality of care [23]. Health information, including health care procedures, birth and mortality registrations from both the public and private sectors, is integrated into the SUS universal information system [23]. The COVID-19 outbreak and response affected maternal and neonatal health services worldwide [2,3]. Several factors, but socioeconomic vulnerability in particular, seem to have exacerbated the impact of the pandemic in Brazil [24]. The COVID-19 pandemic resulted in a decrease of non-COVID health care procedures, such as screenings, diagnostic procedures, physician appointments and low and medium complexity surgeries, despite the increase in human, physical, and financial resources, along with heterogeneous distribution of resources that did not prioritise the most vulnerable states [25].

Regarding stillbirths, in agreement with our findings, other countries around the world have also seen an increase in stillbirths during the COVID-19 pandemic [26,27]. An intense placental inflammatory reaction as a direct consequence of SARS-CoV-2 infection was identified as a possible cause of foetal death [28]. Another study, on more than 1.2 million delivery hospitalisations during March 2020 and September 2021, found that US women who experienced COVID-19 had an almost 2-fold risk for stillbirth compared to women without COVID-19 [29]. However, the increased stillbirths may also have resulted from indirect effects of the pandemic as assumed by Khalil et al. [26]. Their explanation was based on the reluctance to go to the hospital when necessary (for example in a situation of reduced foetal movements) for fear of contagion. In addition, the risk for stillbirths associated with COVID-19 seems to be affected by maternal morbidity, such as chronic hypertension, multiple pregnancies, adverse cardiac event/outcome, placental abruption, sepsis, shock, acute respiratory distress syndrome, mechanical ventilation, and ICU admission, which were associated with a higher prevalence of stillbirths [29].

A recent study including 18 low- and middle-income countries (LMICs) estimated that decreases in essential health service utilisation between March 2020 and June 2021 were associated with 113 962 excess deaths (110 686 children under-5 and 3276 mothers), representing 3.6% and 1.5% increases in child and maternal mortality, respectively [30]. Our findings showed that there was no increase in under-5 deaths after the beginning of the pandemic, but rather, the restrictions appeared to have been protective of children under-five. Over the last few decades, Brazil has made considerable progress in reducing under-5 deaths [9] which seems to have been maintained even during the pandemic. A possible explanatory hypothesis is that some pandemic mitigation measures could have contributed to reduce child exposure to other pathogens that cause disease in the paediatric age group. For example, with school closures there was a reduced number of social contacts and therefore less disease transmission, as well as an increased awareness of hygiene (e.g. hand washing) that may have reduced the contact with pathogens, leading to fewer respiratory tract infections and related respiratory diseases caused by common respiratory viruses, which is one important cause of paediatric deaths [31].

Regarding maternal mortality, prior to the COVID-19 pandemic, Brazil implemented several policies that influenced substantial progress in reducing this indicator since the 1990s [10]. However, problems related to socioeconomic inequalities and lack of quality in the care provided are still challenging, especially considering that the average coverage levels were above 90% for at least four antenatal care visits and skilled birth assistance [32].

To a lesser extent, many countries also reported an increase in maternal mortality after the onset of the pandemic [12,13]. Since maternal deaths from COVID-19 emerged, the scientific community made efforts to explore and understand the clinical features related to mortality among pregnant and postpartum women. In general, studies have shown that pregnant women, especially those with comorbidities including diabetes mellitus, hypertension, and cardiovascular disease, are at increased risk for severe disease from COVID-19, and it is associated with an increased risk for adverse perinatal outcomes [33].

Our results showed that the number of maternal deaths and consequently the MMR suddenly increased after the onset of the pandemic. Maternal mortality is classified by World Health Organization into direct causes (complications related to interventions, omissions, incorrect treatment due to the obstetric state) and indirect causes (resulting from existing diseases or diseases that developed during pregnancy) [34]. During the lasts 20 years the most common causes of maternal deaths were the hypertensive pregnancy diseases, with transition to mortality due to indirect causes, including various conditions from diabetes to infectious diseases [10]. Szwarcwald et al. [35] evaluated COVID-19 related deaths in Brazil in 2020-21 and found that the MMR due to COVID-19 was 35.7 per 100 000 live births. In fact, our results showed that the months with the highest maternal mortality coincided with the second COVID-19 wave, which was more severe and aggressive in Brazil than the first wave [36]. Many of the other non-COVID-19 maternal deaths were probably indirectly related to the pandemic, considering the overload of the health system and the drop of previously mentioned medical procedures (e.g. screening, diagnostic procedures), in addition to efforts to deal with the pandemic being centred around the hospital's human and physical resources instead of preventive actions on primary care [24].

Our results confirmed the existing regional health inequalities in Brazil. The Southeast and South regions, among the richest regions, were characterised by lower maternal and under-5 mortality rates before the pandemic. However, during the pandemic the increase in maternal deaths was highest in the South region, which can be explained by the circulation of new and more contagious variants of the virus, especially in 2021 [36], combined with inadequate sanitary measures to combat the COVID-19 in the South region. On the other hand, the highest reductions in under-5 deaths were observed in the Southeast and South regions. Historically, the North and Northeast regions are more socially vulnerable and have worse health indicators, while people residing in the South and Southeast regions of Brazil have greater access to health services [37].

Other studies may help to understand these regional differences. Siqueira et al. [38] investigated the spatial distribution of COVID-19 cases and maternal deaths in Brazil and their association with social determinants of health. They concluded that COVID-19 cases and deaths in the obstetric population had a heterogeneous geographical distribution, with municipalities with a high degree of socioeconomic dissimilarities showing higher maternal mortality rates than areas with better social and infrastructure indicators [38]. In this context, it is important to highlight that only 15% of the Brazil public maternity hospitals have ICU beds, most of them concentrated in the South, Southeast and Central-West regions [39]. Trying to understand how mitigation measures related to COVID-19 may have differently affected mortality indicators across Brazilian regions is not simple, since in Brazil even federation units of the same region (and also municipalities of each federation unit) were free to adopt different measures with respect to closing schools and borders, social distancing, and lockdowns [40].

Our analyses showed spikes of deaths (especially maternal deaths) occurred when the policy responses to the spread of COVID-19 were strongest, as measured by the stringency index. There are some plausible explanations for this. First, it is possible that the restrictions measures were reinforced as the number of cases (and consequently deaths) was increasing, in order to limit them. It is also possible that, as previously mentioned, with the increase in cases and consequently in the restrictions imposed to contain the pandemic, the fear of attending health services also increased leding to a delay in identifying and treating important risk health situations during pregnancy that required more urgent care. On the other hand, as in the study by Chmielewska et al. [12], this finding may also suggest that the increased adverse outcomes may be mainly due to an inability to deal with the pandemic and problems inherent to health services rather than to the imposed mitigation measures.

This study is prone to limitations associated with the use of routine health services data, which include challenges related to under-reporting of deaths (which already existed prior to the pandemic) [10] and the fact of using preliminary mortality data from 2021. However, it should be noted that studies have shown improvements in the quality of information on births and deaths from the health system in Brazil, found most data coverage values above 90% [41,42]. Furthermore, U5MR and MMR until 2020 were calculated by the Ministry of Health using correction factors, considering the existence of underreporting. For 2021, the values were not corrected yet, but usually the values increase after the adjustments [41]. Thus, it is likely that any change in mortality data due to future corrections may have little influence on our results. Finally, COVID-19 has disrupted health systems, and as a result, estimates might have been affected. Nevertheless, we utilised sequential data from each region, which provides the most accurate estimate of the situation. Additionally, by using time series, we were able to observe patterns effectively.

The strengths of this study include the use of national data, the inclusion of a comparable period of three previous years and a strong technique to estimate monthly changes in each type of death that accounted for historical trends, seasonality, and population growth. In addition, we disaggregated the analysis by regional level, which allowed us to identify distinct mortality patterns in Brazil’s regions.

## CONCLUSIONS

The COVID-19 pandemic highlighted the Brazilian socioeconomic and health system vulnerabilities. There were many problems in the management of the pandemic in Brazil that undoubtedly worsened the situation, such as the delay in adopting public health measures and in acquiring vaccines for controlling the spread of the pandemic [35], which resulted in several avoidable deaths. Our results emphasise the pandemic’s impact on mortality trends, with an increase in stillbirths, a substantial increase in maternal deaths and MMR with important regional differences, and a decrease in under-5 deaths compared to the expected numbers of deaths based on the months prior to the pandemic. To mitigate the effects of reduced access to health care in vulnerable regions, it is essential to develop strategies and direct resources towards strategic support policies for preventing the main risk factors in priority populations, as these interventions can have a significant impact. Concerning maternal mortality, there is an emphasis on prioritising reproductive health care and promptly creating new models of women-centred obstetric care throughout the country. This approach aims to improve preparedness and response to health emergencies more effectively.

Our findings reinforce the importance of focusing on maternal and child health, especially in most remote regions, and may contribute to planning specific actions to re-establish pre-pandemic efforts to achieve the SDGs. Despite its relevance, stillbirths are not included by the United Nations as one of the SDGs, deserving a special attention in the global policy agenda.

## Additional material


Online Supplementary Document
