# Supplementary material for: The impact of the COVID-19 pandemic on trends in stillbirths, under-5 and maternal mortality in Brazil: Excess deaths and regional inequalities
Source: J Glob Health. 2023 Sep 30;13:06040. doi: 10.7189/jogh.13.06040 (PMC10540663; doi:10.7189/jogh.13.06040)
Supplement: Online Supplementary Document [file jogh-13-06040-s001.pdf]

Supplementary material

The impact of the COVID-19 pandemic on trends in stillbirths, under-5 and maternal mortality in Brazil: Excess deaths and health inequalities

Table S1. Percentage change in stillbirth, under-5 deaths and maternal deaths by month from March 2020 to December 2021, compared to the expected deaths based on the preceding three years.

Table S2. Percentage change in stillbirth, under-5 deaths and maternal deaths by month and region in Brazil from March 2020 to December 2021, compared to the expected deaths based on the preceding three years.

Table S3. Observed and expected values of maternal mortality ratio (MMR) and under-5 mortality rate (U5MR) in 2020 and 2021 by region in Brazil

Table S1. Percentage change in stillbirth, under-5 deaths and maternal deaths by month from March 2020 to December 2021, compared to the expected deaths based on the preceding three years.

Timepoints (month year)	Observed	Expected	Observed Lower bound	Observed Upper bound	Expected Lower bound	Expected Upper bound	% change
**Stillbirths**							
**Mar 2020**	2532	2546.01	1975.30	3088.70	1978.08	3113.93	-0.55
**Apr 2020**	2542	2487.34	1984.77	3099.23	1918.45	3056.23	2.20
**May 2020**	2628	2555.01	2070.23	3185.77	1985.13	3124.89	2.86
**Jun 2020**	2442	2296.01	1883.68	3000.32	1725.11	2866.90	6.36
**Jul 2020**	2552	2289.67	1993.12	3110.89	1717.74	2861.60	11.46
**Aug 2020**	2402	2292.34	1842.54	2961.46	1719.35	2865.32	4.78
**Sep 2020**	2304	2141.34	1743.95	2864.05	1567.28	2715.40	7.60
**Oct 2020**	2204	2285.67	1643.35	2764.66	1710.51	2860.84	-3.57
**Nov 2020**	2256	2257.01	1694.73	2817.27	1680.72	2833.29	-0.04
**Dec 2020**	2327	2320.01	1765.10	2888.90	1742.58	2897.44	0.30
**2020 TOTAL**	**24189**	**23470.39**					**3.06**
**Jan 2021**	2579	2502.70	2016.02	3141.98	1923.78	3081.62	3.05
**Feb 2021**	2294	2257.70	1730.37	2857.63	1677.59	2837.81	1.61
**Mar 2021**	2761	2473.20	2196.71	3325.29	1889.39	3057.02	11.64
**Apr 2021**	2543	2414.54	1978.03	3107.97	1829.50	2999.58	5.32
**May 2021**	2514	2482.20	1948.34	3079.66	1895.92	3068.49	1.28
**Jun 2021**	2427	2223.20	1860.64	2993.36	1635.66	2810.75	9.17
**Jul 2021**	2371	2216.87	1803.93	2938.07	1628.04	2805.70	6.95
**Aug 2021**	2397	2219.54	1829.20	2964.80	1629.40	2809.67	8.00
**Sep 2021**	2326	2068.54	1757.47	2894.53	1477.08	2659.99	12.45
**Oct 2021**	2347	2212.87	1777.72	2916.28	1620.07	2805.67	6.06
**Nov 2021**	2301	2184.20	1730.96	2871.04	1590.04	2778.37	5.35
**Dec 2021**	2350	2247.20	1779.19	2920.82	1651.66	2842.75	4.57
**2021 TOTAL**	**29210**	**27502.77**					**6.21**
**TOTAL COVID PERIOD**	**53399**	**50973.16**					**4.76**

**Under-5 deaths**							
**Mar 2020**	3424	3585.24	2808.69	4039.31	3025.01	4145.47	-4.50
**Apr 2020**	3074	3589.24	2455.65	3692.35	3028.28	4150.20	-14.36
**May 2020**	3180	3721.91	2558.54	3801.46	3160.21	4283.61	-14.56
**Jun 2020**	2860	3475.91	2235.38	3484.62	2913.45	4038.37	-17.72
**Jul 2020**	2849	3432.57	2221.17	3476.83	2869.33	3995.81	-17.00
**Aug 2020**	2895	3286.91	2263.89	3526.11	2722.87	3850.94	-11.92
**Sep 2020**	2727	3112.57	2092.56	3361.44	2547.73	3677.42	-12.39
**Oct 2020**	2922	3211.91	2284.18	3559.83	2646.23	3777.58	-9.03
**Nov 2020**	2751	3210.24	2109.74	3392.26	2643.72	3776.76	-14.31
**Dec 2020**	3044	3354.57	2399.24	3688.76	2787.19	3921.95	-9.26
**2020 TOTAL**	**29726**	**33981.06**					**-12.52**
**Jan 2021**	3139	3271.68	2489.68	3788.32	2703.16	3840.19	-4.06
**Feb 2021**	2852	3024.68	2199.09	3504.91	2455.27	3594.09	-5.71
**Mar 2021**	3251	3532.24	2594.46	3907.54	2958.09	4106.38	-7.96
**Apr 2021**	2980	3536.24	2319.77	3640.23	2961.17	4111.30	-15.73
**May 2021**	3099	3668.90	2435.04	3762.96	3092.91	4244.90	-15.53
**Jun 2021**	2974	3422.90	2306.26	3641.74	2845.95	3999.85	-13.11
**Jul 2021**	3165	3379.57	2493.43	3836.57	2801.65	3957.49	-6.35
**Aug 2021**	2954	3233.90	2278.56	3629.44	2655.00	3812.81	-8.66
**Sep 2021**	2852	3059.57	2172.64	3531.36	2479.67	3639.47	-6.78
**Oct 2021**	3075	3158.90	2391.68	3758.32	2577.99	3739.82	-2.66
**Nov 2021**	2998	3157.24	2310.68	3685.32	2575.29	3739.19	-5.04
**Dec 2021**	3177	3301.57	2485.63	3868.37	2718.58	3884.56	-3.77
**2021 TOTAL**	**36516**	**39747.39**					**-8.13**
**TOTAL COVID PERIOD**	**66242**	**73728.45**					**-10.15**
**Maternal deaths**							
**Mar 2020**	149	145.96	96.29	201.71	108.54	183.39	2.08
**Apr 2020**	176	135.96	123.15	228.85	98.41	173.52	29.45
**May 2020**	252	145.30	199.01	304.99	107.62	182.98	73.44
**Jun 2020**	208	134.96	154.86	261.14	97.15	172.78	54.11
**Jul 2020**	201	124.30	147.72	254.28	86.35	162.24	61.71

**Aug 2020**	154	121.63	100.57	207.43	83.55	159.71	26.61
**Sep 2020**	135	117.30	81.41	188.59	79.08	155.52	15.09
**Oct 2020**	149	108.30	95.26	202.74	69.94	146.66	37.58
**Nov 2020**	142	108.30	88.10	195.90	69.79	146.80	31.12
**Dec 2020**	145	122.30	90.94	199.06	83.65	160.95	18.56
**2020 TOTAL**	**1711**	**1264.32**					**35.33**
**Jan 2021**	197	127.99	145.67	248.33	89.23	166.75	53.92
**Feb 2021**	193	111.49	141.49	244.51	72.58	150.40	73.11
**Mar 2021**	392	140.23	340.30	443.70	99.84	180.63	179.53
**Apr 2021**	454	130.23	402.12	505.88	89.69	170.78	248.61
**May 2021**	388	139.57	335.93	440.07	98.87	180.27	178.00
**Jun 2021**	368	129.23	315.74	420.26	88.38	170.09	184.76
**Jul 2021**	247	118.57	194.54	299.46	77.55	159.58	108.32
**Aug 2021**	169	115.90	116.35	221.65	74.73	157.07	45.82
**Sep 2021**	149	111.57	96.15	201.85	70.23	152.90	33.55
**Oct 2021**	128	102.57	74.94	181.06	61.07	144.07	24.80
**Nov 2021**	135	102.57	81.74	188.26	60.90	144.23	31.62
**Dec 2021**	121	116.57	67.53	174.47	74.73	158.40	3.80
**2021 TOTAL**	**2941**	**1446.48**					**103.32**
**TOTAL COVID PERIOD**	**4652**	**2710.67**					**71.62**

Table S2. Percentage change in stillbirth, under-5 deaths and maternal deaths by month and region in Brazil from March 2020 to December 2021, compared to the expected deaths based on the preceding three years.

Timepoints (month year)	Stillbirths			Under-5 deaths			Maternal deaths		
	Observed (lower and upper bounds)	Expected (lower and upper bounds)	% change	Observed (lower and upper bounds)	Expected (lower and upper bounds)	% change	Observed (lower and upper bounds)	Expected (lower and upper bounds)	% change
**North**									
Mar 2020	306 (253.6; 358.4)	279.0 (236.2; 321.9)	9.66	471 (363.0; 579.0)	499.8 (407.6; 592.0)	-5.76	15 (1.7; 28.3)	17.6 (11.1; 24.2)	-14.89
Apr 2020	290 (237.5; 342.5)	311.7 (268.8; 354.6)	-6.96	411 (302.6; 519.4)	515.1 (422.7; 607.6)	-20.22	29 (15.7; 42.3)	18.0 (11.4; 24.5)	61.49
May 2020	389 (336.5; 441.5)	323.7 (280.8; 366.6)	20.17	412 (303.2; 520.8)	537.8 (445.1; 630.5)	-23.39	48 (34.7; 61.3)	22.3 (15.7; 28.9)	115.33
Jun 2020	300 (247.5; 352.5)	290.4 (247.4; 333.3)	3.31	424 (314.7; 533.3)	493.5 (400.4; 586.5)	-14.08	29 (15.6; 42.4)	17.3 (10.7; 23.9)	67.72
Jul 2020	329 (276.5; 381.5)	292.0 (249.1; 335.0)	12.65	422 (312.3; 531.7)	480.1 (386.8; 573.5)	-12.11	24 (10.6; 37.4)	19.0 (12.3; 25.6)	26.60
Aug 2020	288 (235.5; 340.5)	313.7 (270.7; 356.7)	-8.20	424 (313.8; 534.2)	483.5 (389.8; 577.1)	-12.30	22 (8.6; 35.4)	24.3 (17.6; 30.9)	-9.43
Sep 2020	296 (243.5; 348.5)	275.0 (232.0; 318.1)	7.62	400 (289.4; 510.6)	454.8 (360.8; 548.8)	-12.05	22 (8.6; 35.4)	19.3 (12.6; 26.0)	14.04
Oct 2020	238 (185.4; 290.6)	294.7 (251.6; 337.8)	-19.24	427 (315.9; 538.1)	446.8 (352.5; 541.1)	-4.43	28 (14.5; 41.5)	16.0 (9.3; 22.7)	75.47
Nov 2020	246 (193.4; 298.6)	287.0 (243.9; 330.2)	-14.30	421 (309.4; 532.6)	474.8 (380.2; 569.4)	-11.33	16 (2.5; 29.5)	20.0 (13.2; 26.7)	-19.83
Dec 2020	285 (232.4; 337.6)	312.7 (269.5; 355.9)	-8.86	466 (353.9; 578.1)	449.8 (354.9; 544.8)	3.60	17 (3.5; 30.5)	16.3 (9.6; 23.0)	4.35
**2020 TOTAL**	**2967**	**2980.1**	**-0.44**	**4278**	**4836.08**	**-11.54**	**250**	**189.91**	**31.64**
Jan 2021	331 (277.7; 384.3)	314.5 (271.3; 357.8)	5.23	485 (372.2; 597.8)	446.4 (350.9; 541.9)	8.65	43 (30.1; 55.9)	19.1 (12.4; 25.8)	125.13
Feb 2021	294 (240.7; 347.3)	289.8 (246.5; 333.1)	1.45	422 (308.7; 535.3)	439.4 (343.6; 535.2)	-3.96	55 (42.1; 67.9)	15.8 (9.1; 22.6)	247.01
Mar 2021	343 (289.7; 396.3)	280.5 (234.6; 326.5)	22.26	450 (336.1; 563.9)	499.1 (400.5; 597.6)	-9.84	61 (48.1; 73.9)	17.3 (9.6; 24.9)	253.51
Apr 2021	337 (283.6; 390.4)	313.2 (267.3; 359.2)	7.59	432 (317.6; 546.4)	514.4 (415.5; 613.3)	-16.02	46 (33.0; 59.0)	17.6 (9.9; 25.3)	161.53
May 2021	303 (249.6; 356.4)	325.2 (279.2; 371.2)	-6.83	419 (304.1; 533.9)	537.1 (437.8; 636.3)	-21.99	44 (31.0; 57.0)	21.9 (14.2; 29.6)	100.71
Jun 2021	284 (230.6; 337.4)	291.9 (245.8; 337.9)	-2.70	391 (275.6; 506.4)	492.8 (393.2; 592.4)	-20.65	41 (28.0; 54.0)	16.9 (9.2; 24.7)	142.28
Jul 2021	275 (221.6; 328.4)	293.5 (247.5; 339.6)	-6.32	473 (357.0; 589.0)	479.4 (379.5; 579.4)	-1.34	32 (18.9; 45.1)	18.6 (10.8; 26.4)	72.14
Aug 2021	297 (243.5; 350.5)	315.2 (269.1; 361.4)	-5.78	467 (350.5; 583.5)	482.8 (382.4; 583.1)	-3.26	24 (10.9; 37.1)	23.9 (16.1; 31.7)	0.32
Sep 2021	297 (243.5; 350.5)	276.5 (230.4; 322.7)	7.40	464 (346.9; 581.1)	454.1 (353.4; 554.8)	2.18	23 (9.9; 36.1)	18.9 (11.1; 26.7)	21.55
Oct 2021	308 (254.5; 361.5)	296.2 (250.0; 342.5)	3.98	469 (351.4; 586.6)	446.1 (345.0; 547.2)	5.14	25 (11.8; 38.2)	15.6 (7.8; 23.4)	60.37
Nov 2021	289 (235.5; 342.5)	288.5 (242.3; 334.8)	0.16	436 (317.8; 554.2)	474.1 (372.6; 575.6)	-8.03	22 (8.8; 35.2)	19.6 (11.7; 27.5)	12.31
Dec 2021	319 (265.4; 372.6)	314.2 (267.9; 360.5)	1.52	466 (347.2; 584.8)	449.1 (347.2; 550.9)	3.76	18 (4.7; 31.3)	15.9 (8.0; 23.8)	13.05
**2021 TOTAL**	**3677**	**3599.47**	**2.15**	**5374**	**5714.71**	**-5.96**	**434**	**221.17**	**96.23**

**TOTAL COVID PERIOD**	**6644**	**6579.57**	**0.98**	**9652**	**10550.79**	**-8.52**	**684**	**411.08**	**66.39**
**Northeast**									
Mar 2020	849 (721.4; 976.6)	819.72 (679.7; 959.7)	3.57	1147 (980.8; 1313.2)	1102.2 (957.7; 1246.7)	4.07	57 (37.4; 76.6)	42.3 (30.4; 54.2)	34.78
Apr 2020	859 (731.2; 986.8)	838.05 (697.2; 978.9)	2.50	989 (821.9; 1156.1)	1122.9 (978.0; 1267.7)	-11.92	68 (48.3; 87.7)	48.3 (36.4; 60.2)	40.81
May 2020	895 (767.0; 1023.0)	823.39 (681.6; 965.1)	8.70	1059 (891.1; 1226.9)	1150.2 (1004.9; 1295.5)	-7.93	96 (76.3; 115.7)	47.0 (35.0; 58.9)	104.43
Jun 2020	868 (739.8; 996.2)	756.72 (614.1; 899.3)	14.71	943 (774.2; 1111.8)	1070.9 (925.2; 1216.5)	-11.94	77 (57.2; 96.8)	43.3 (31.3; 55.2)	77.86
Jul 2020	863 (734.6; 991.4)	792.39 (648.9; 935.9)	8.91	923 (753.2; 1092.8)	1040.2 (894.2; 1186.2)	-11.27	65 (45.2; 84.8)	32.0 (20.0; 43.9)	103.38
Aug 2020	853 (724.4; 981.6)	768.39 (624.0; 912.8)	11.01	928 (757.3; 1098.7)	1007.9 (861.4; 1154.3)	-7.92	49 (29.2; 68.8)	38.6 (26.6; 50.6)	26.86
Sep 2020	752 (623.2; 880.8)	744.72 (599.4; 890.1)	0.98	854 (682.4; 1025.6)	936.5 (789.7; 1083.4)	-8.81	38 (18.1; 57.9)	32.6 (20.6; 44.7)	16.47
Oct 2020	729 (600.0; 858.0)	735.39 (589.1; 881.7)	-0.87	864 (691.4; 1036.6)	990.5 (843.3; 1137.8)	-12.77	51 (31.1; 70.9)	29.6 (17.6; 41.7)	72.15
Nov 2020	712 (582.8; 841.2)	756.05 (608.8; 903.3)	-5.83	858 (684.4; 1031.6)	979.5 (831.8; 1127.2)	-12.41	42 (22.0; 62.0)	32.0 (19.9; 44.0)	31.42
Dec 2020	713 (583.5; 842.5)	732.72 (584.5; 880.9)	-2.69	883 (708.4; 1057.6)	1034.5 (886.4; 1182.7)	-14.65	42 (22.0; 62.0)	37.0 (24.8; 49.1)	13.64
**2020 TOTAL**	**8093**	**7767.52**	**4.19**	**9448**	**10435.26**	**-9.46**	**585**	**382.59**	**52.90**
Jan 2021	841 (711.1; 970.9)	791.6 (642.4; 940.8)	6.24	926 (749.7; 1102.3)	996.0 (847.3; 1144.6)	-7.02	48 (29.0; 67.0)	36.1 (24.0; 48.2)	32.94
Feb 2021	751 (620.8; 881.2)	699.8 (549.6; 850.0)	7.31	892 (714.7; 1069.3)	936.0 (786.9; 1085.0)	-4.70	46 (27.0; 65.0)	34.4 (22.2; 46.5)	33.89
Mar 2021	910 (779.6; 1040.4)	790.0 (636.1; 943.9)	15.19	1025 (846.6; 1203.4)	1076.0 (923.0; 1229.0)	-4.74	86 (66.9; 105.1)	39.9 (26.6; 53.2)	115.38
Apr 2021	825 (694.4; 955.6)	808.3 (653.4; 963.2)	2.06	982 (802.6; 1161.4)	1096.7 (943.2; 1250.1)	-10.46	131 (111.8; 150.1)	45.9 (32.6; 59.3)	185.23
May 2021	851 (720.1; 981.9)	793.7 (637.8; 949.6)	7.22	1090 (909.5; 1270.5)	1124.0 (970.1; 1277.9)	-3.03	110 (90.8; 129.2)	44.6 (31.2; 58.0)	146.66
Jun 2021	828 (696.9; 959.1)	727.0 (570.1; 883.9)	13.89	988 (806.5; 1169.5)	1044.7 (890.3; 1199.1)	-5.43	101 (81.8; 120.2)	40.9 (27.5; 54.3)	146.77
Jul 2021	813 (681.6; 944.4)	762.7 (604.7; 920.6)	6.60	943 (760.4; 1125.6)	1014.0 (859.1; 1168.9)	-7.00	92 (72.7; 111.3)	29.6 (16.2; 43.0)	210.86
Aug 2021	749 (617.3; 880.7)	738.7 (579.7; 897.7)	1.40	946 (762.3; 1129.7)	981.7 (826.3; 1137.0)	-3.63	45 (25.6; 64.4)	36.3 (22.8; 49.7)	24.10
Sep 2021	740 (608.1; 871.9)	715.0 (554.9; 875.1)	3.50	853 (668.2; 1037.8)	910.3 (754.5; 1066.2)	-6.30	35 (15.6; 54.4)	30.3 (16.8; 43.7)	15.66
Oct 2021	782 (649.8; 914.2)	705.7 (544.5; 866.8)	10.82	996 (810.1; 1181.9)	964.3 (808.0; 1120.7)	3.28	38 (18.5; 57.5)	27.3 (13.7; 40.8)	39.39
Nov 2021	757 (624.5; 889.5)	726.3 (564.1; 888.6)	4.22	903 (716.0; 1090.0)	953.3 (796.5; 1110.2)	-5.28	45 (25.4; 64.6)	29.6 (16.0; 43.1)	52.05
Dec 2021	710 (577.3; 842.7)	703.0 (539.7; 866.3)	1.00	909 (720.8; 1097.2)	1008.3 (851.0; 1165.7)	-9.85	41 (21.4; 60.6)	34.6 (21.0; 48.2)	18.51
**2021 TOTAL**	**9557**	**8961.76**	**6.64**	**11453**	**12105.38**	**-5.39**	**818**	**429.4**	**90.49**
**TOTAL COVID PERIOD**	**17650**	**16729.28**	**5.50**	**20901**	**22540.64**	**-7.27**	**1403**	**811.99**	**72.79**
**Southeast**									

Mar 2020	949 (760.6; 1137.4)	958.4 (785.3; 1131.5)	-0.98	1208 (986.4; 1429.6)	1273.9 (1152.3; 1395.6)	-5.18	54 (23.1; 84.9)	60.0 (44.5; 75.4)	-9.93
Apr 2020	914 (725.2; 1102.8)	885.4 (711.8; 1059.0)	3.23	1087 (861.7; 1312.3)	1265.9 (1143.2; 1388.7)	-14.13	62 (31.0; 93.0)	46.6 (31.1; 62.2)	32.99
May 2020	938 (748.7; 1127.3)	957.1 (783.0; 1131.2)	-1.99	1101 (871.9; 1330.1)	1358.9 (1235.1; 1482.7)	-18.98	82 (50.9; 113.1)	47.6 (32.0; 63.3)	72.20
Jun 2020	845 (655.3; 1034.7)	841.1 (666.4; 1015.7)	0.47	954 (721.1; 1186.9)	1272.6 (1147.7; 1397.5)	-25.04	63 (31.8; 94.2)	45.6 (29.9; 61.3)	38.10
Jul 2020	922 (731.8; 1112.2)	817.1 (641.9; 992.2)	12.84	956 (719.3; 1192.7)	1217.3 (1091.2; 1343.3)	-21.46	68 (36.7; 99.3)	47.0 (31.2; 62.7)	44.83
Aug 2020	855 (664.4; 1045.6)	802.7 (627.0; 978.5)	6.51	978 (737.4; 1218.6)	1152.6 (1025.4; 1279.8)	-15.15	47 (15.5; 78.5)	37.6 (21.7; 53.5)	24.94
Sep 2020	820 (628.9; 1011.1)	755.1 (578.8; 931.3)	8.60	929 (684.5; 1173.5)	1098.9 (970.6; 1227.2)	-15.46	57 (25.4; 88.6)	44.3 (28.3; 60.2)	28.71
Oct 2020	821 (629.4; 1012.6)	839.7 (662.9; 1016.6)	-2.23	1050 (801.6; 1298.4)	1135.3 (1005.8; 1264.7)	-7.51	46 (14.3; 77.7)	39.0 (22.9; 55.0)	18.09
Nov 2020	828 (635.9; 1020.1)	818.7(641.3; 996.1)	1.13	988 (735.7; 1240.3)	1148.6 (1018.0; 1279.2)	-13.98	53 (21.2; 84.8)	36.3 (20.2; 52.4)	46.06
Dec 2020	898 (705.4; 1090.6)	864.7 (686.7; 1042.7)	3.85	1098 (841.8; 1354.2)	1232.6 (1100.8; 1364.4)	-10.92	56 (24.1; 87.9)	49.6 (33.4; 65.8)	12.86
**2020 TOTAL**	**8790**	**8540.1**	**2.93**	**10349**	**12156.62**	**-14.87**	**588**	**453.53**	**29.65**
Jan 2021	946 (752.6; 1139.4)	953.7 (774.8; 1132.7)	-0.81	1161 (899.5; 1422.5)	1182.1 (1049.2; 1315.1)	-1.79	68 (42.2; 93.8)	46.5 (30.2; 62.8)	46.22
Feb 2021	832 (638.1; 1025.9)	843.5 (663.9; 1023.0)	-1.36	968 (702.6; 1233.4)	1068.6 (934.5; 1202.8)	-9.42	64 (38.0; 90.0)	38.3 (21.9; 54.6)	67.30
Mar 2021	994 (799.6; 1188.4)	927.3 (744.5; 1110.1)	7.19	1149 (879.6; 1418.4)	1255.1 (1114.1; 1396.1)	-8.45	139 (112.9; 165.1)	56.7 (39.2; 74.3)	144.96
Apr 2021	916 (721.0; 1111.0)	854.3 (670.9; 1037.7)	7.22	1006 (732.6; 1279.4)	1247.1 (1104.9; 1389.3)	-19.33	182 (155.8; 208.2)	43.4 (25.8; 61.0)	319.26
May 2021	906 (710.5; 1101.5)	926.0 (741.9; 1110.0)	-2.16	1008 (730.6; 1285.4)	1340.1 (1196.7; 1483.5)	-24.78	151 (124.6; 177.4)	44.4 (26.7; 62.1)	240.01
Jun 2021	870 (673.9; 1066.1)	810.0 (625.3; 994.7)	7.41	994 (712.5; 1275.5)	1253.8 (1109.2; 1398.4)	-20.72	119 (92.5; 145.5)	42.4 (24.6; 60.2)	180.59
Jul 2021	841 (644.4; 1037.6)	786.0 (600.7; 971.3)	7.00	1093 (807.5; 1378.5)	1198.4 (1052.6; 1344.3)	-8.80	64 (37.3; 90.7)	43.7 (25.9; 61.6)	46.31
Aug 2021	883 (685.8; 1080.2)	771.6 (585.7; 957.6)	14.43	998 (708.4; 1287.6)	1133.8 (986.7; 1280.8)	-11.98	56 (29.2; 82.8)	34.4 (16.4; 52.4)	62.74
Sep 2021	878 (680.2; 1075.8)	724.0 (537.3; 910.6)	21.28	938 (644.3; 1231.7)	1080.1 (931.8; 1228.4)	-13.16	55 (28.0; 82.0)	41.1 (23.0; 59.1)	33.90
Oct 2021	774 (575.6; 972.4)	808.6 (621.3; 995.9)	-4.28	1018 (720.2; 1315.8)	1116.4 (966.9; 1266.0)	-8.82	35 (7.8; 62.2)	35.7 (17.6; 53.9)	-2.08
Nov 2021	838 (639.0; 1037.0)	787.6 (599.7; 975.6)	6.39	1033 (731.1; 1334.9)	1129.8 (978.9; 1280.6)	-8.57	43 (15.7; 70.3)	33.1 (14.8; 51.3)	30.00
Dec 2021	900 (700.4; 1099.6)	833.6 (645.0; 1022.3)	7.96	1179 (873.0; 1485.0)	1213.8 (1061.6; 1365.9)	-2.87	37 (9.5; 64.5)	46.4 (28.1; 64.8)	-20.28
**2021 TOTAL**	**10578**	**10026.2**	**5,5**	**12545**	**14219.18**	**-11.77**	**1013**	**506.19**	**100.12**
**TOTAL COVID PERIOD**	**19368**	**18566.26**	**4,32**	**22894**	**26375.8**	**-13.20**	**1601**	**959.72**	**66.82**
**South**									
Mar 2020	228 (197.3; 258.7)	288.1 (266.3; 309.9)	-20.86	308 (248.7; 367.3)	391.6 (346.5; 436.6)	-21.34	9 (-5.2; 23.2)	11.16 (6.2; 16.1)	-19.38
Apr 2020	281 (250.3; 311.7)	255.1 (233.3; 276.9)	10.16	322 (262.5; 381.5	370.2 (325.2; 415.3)	-13.03	11 (-3.2; 25.2)	12.2 (7.2; 17.1)	-9.57
May 2020	221 (190.3; 251.7)	257.1 (235.3; 278.9)	-14.04	341 (281.3; 400.7)	390.6 (345.5; 435.6)	-12.69	13 (-1.2; 27.2)	12.5 (7.6; 17.4)	4.03
Jun 2020	220 (189.3; 250.7)	234.4 (212.6; 256.2)	-6.15	310 (250.1; 369.9)	375.9 (330.9; 420.9)	-17.53	15 (0.7; 29.3)	12.2 (7.2; 17.1)	23.32

Jul 2020	240 (209.3; 270.7)	225.7 (203.9; 247.6)	6.31	300 (239.9; 360.1)	406.2 (361.2; 451.3)	-26.15	20 (5.7; 34.3)	13.8 (8.9; 18.8)	44.61
Aug 2020	225 (194.3; 255.7)	223.1 (201.3; 244.9)	0.86	337 (276.7; 397.3)	365.9 (320.9; 410.9)	-7.90	15 (0.7; 29.3)	12.2 (7.2; 17.1)	23.32
Sep 2020	226 (195.3; 256.7)	204.1 (182.3; 225.9)	10.74	297 (236.5; 357.5)	354.6 (309.5; 399.6)	-16.24	7 (-7.4; 21.4)	9.8 (4.9; 14.8)	-28.79
Oct 2020	226 (195.3; 256.7)	229.7 (207.9; 251.6)	-1.63	324 (263.3; 384.7)	364.6 (319.5; 409.6)	-11.13	9 (-5.4; 23.4)	12.2 (7.2; 17.1)	-26.01
Nov 2020	272 (241.3; 302.7)	224.1 (202.3; 245.9)	21.38	285 (224.1; 345.9)	344.6 (299.5; 389.6)	-17.29	21 (6.6; 35.4)	9.5 (4.6; 14.4)	121.13
Dec 2020	235 (204.3; 265.7)	233.4 (211.6; 255.2)	0.68	346 (284.8; 407.2)	362.9 (317.9; 407.9)	-4.66	23 (8.5; 37.5)	8.2 (3.2; 13.1)	181.74
**2020 TOTAL**	**2374**	**2374.8**	**-0.03**	**3170**	**3727.07**	**-14.95**	**143**	**113.64**	**25.84**
Jan 2021	264 (232.5; 295.5)	256.9 (235.3; 278.6)	2.74	331 (268.4; 393.6)	383.1 (338.3; 427.9)	-13.60	18 (6.2; 29.8)	12.9 (8.0; 17.8)	39.89
Feb 2021	246 (214.5; 277.5)	247.9 (226.3; 269.6)	-0.79	309 (246.2; 371.8)	336.1 (291.3; 380.9)	-8.06	13 (1.1; 24.9)	10.6 (5.7; 15.5)	22.45
Mar 2021	273 (241.5; 304.5)	279.7 (254.5; 304.9)	-2.40	338 (275.0; 401.0)	385.7 (338.2; 433.3)	-12.38	57 (45.0; 69.0)	10.9 (5.2; 16.6)	422.57
Apr 2021	259 (227.5; 290.5)	246.7 (221.5; 271.9)	4.98	319 (255.7; 382.3)	364.4 (316.9; 411.9)	-12.46	40 (28.0; 52.0)	11.9 (6.2; 17.6)	235.92
May 2021	249 (217.5; 280.5)	248.7 (223.5; 273.9)	0.11	323 (259.5; 386.5)	384.7 (337.2; 432.3)	-16.05	48 (35.9; 60.1)	12.2 (6.5; 17.9)	292.12
Jun 2021	245 (213.5; 276.5)	226.1 (200.8; 251.3)	8.38	349 (285.3; 412.7)	370.1 (322.5; 417.6)	-5.69	72 (59.8; 84.2)	11.9 (6.2; 17.6)	504.65
Jul 2021	211 (179.5; 242.5)	217.4 (192.2; 242.6)	-2.94	364 (300.1; 427.9)	400.4 (352.9; 447.9)	-9.09	35 (22.8; 47.2)	13.6 (7.8; 19.3)	157.84
Aug 2021	263 (231.5; 294.5)	214.7 (189.5; 239.9)	22.49	318 (253.8; 382.2)	360.1 (312.5; 407.6)	-11.68	23 (10.7; 35.3)	11.9 (6.2; 17.6)	93.15
Sep 2021	219 (187.5; 250.5)	195.7 (170.5; 220.9)	11.90	324 (259.6; 388.4)	348.7 (301.2; 396.3)	-7.09	27 (14.6; 39.4)	9.6 (3.9; 15.3)	182.00
Oct 2021	284 (252.5; 315.5)	221.4 (196.2; 246.6)	28.28	334 (269.4; 398.6)	358.7 (311.2; 406.3)	-6.90	17 (4.6; 29.4)	11.9 (6.2; 17.6)	42.76
Nov 2021	235 (203.5; 266.5)	215.7 (190.5; 240.9)	8.94	340 (275.1; 404.9)	338.7 (291.2; 386.3)	0.37	17 (4.5; 29.5)	9.2 (3.5; 14.9)	83.96
Dec 2021	236 (204.5; 267.5)	225.1 (199.8; 250.3)	4.86	337 (271.9; 402.1)	357.1 (309.5; 404.6)	-5.62	14 (1.4; 26.6)	7.9 (2.2; 13.6)	77.04
**2021 TOTAL**	**2984**	**2796.09**	**6.72**	**3986**	**4387.87**	**-9.16**	**381**	**134.56**	**183.14**
**TOTAL COVID PERIOD**	**5358**	**5170.9**	**3.62**	**7156**	**8114.94**	**-11.82**	**524**	**248.2**	**111.12**
**Central-West**									
Mar 2020	200 (165.0; 235.0)	200.7 (172.0; 229.4)	-0.35	290 (233.0; 347.0)	317.6 (266.4; 368.9)	-8.70	14 (3.3; 24.7)	14.9 (9.3; 20.6)	-6.23
Apr 2020	198 (162.9; 233.1)	197.0 (168.1; 226.0)	0.48	265 (207.8; 322.2)	315.0 (263.5; 366.4)	-15.87	6 (-4.8; 16.8)	10.9 (5.2; 16.6)	-45.10
May 2020	185 (149.9; 220.1)	193.7 (164.5; 222.9)	-4.50	267 (209.7; 324.3)	284.3 (232.7; 336.0)	-6.09	13 (2.2; 23.8)	15.9 (10.2; 21.7)	-18.39
Jun 2020	209 (173.8; 244.2)	173.4 (144.0; 202.8)	20.55	229 (171.6; 286.4)	263.0 (211.1; 314.9)	-12.92	24 (13.1; 34.9)	16.6 (10.8; 22.4)	44.61
Jul 2020	198 (162.7; 233.3)	162.4 (132.7; 192.0)	21.94	248 (190.5; 305.5)	288.6 (236.6; 340.7)	-14.08	24 (13.1; 34.9)	12.6 (6.8; 18.4)	90.53
Aug 2020	181 (145.7; 216.3)	184.4 (154.5; 214.3)	-1.83	228 (170.4; 285.7)	277.0 (224.7; 329.3)	-17.68	21 (10.1; 31.9)	8.9 (3.1; 14.8)	135.17
Sep 2020	210 (174.6; 245.4)	162.4 (132.2; 192.5)	29.33	247 (189.2; 304.8)	267.6 (215.1; 320.2)	-7.71	11 (0.0; 22.0)	11.3 (5.4; 17.2)	-2.34
Oct 2020	190 (154.5; 225.5)	186.0 (155.7; 216.4)	2.13	257 (199.1; 314.9)	274.6 (221.9; 327.4)	-6.43	15 (4.0; 26.0)	11.6 (5.6; 17.5)	29.35

Nov 2020	198 (162.4; 233.6)	171.0 (140.4; 201.7)	15.76	199 (141.0; 257.0)	262.6 (209.6; 315.7)	-24.23	10 (-1.1; 21.1)	10.6 (4.6; 16.6)	-5.63
Dec 2020	196 (160.3; 231.7)	176.4 (145.5; 207.3)	11.12	251 (192.8; 309.2)	274.6 (221.4; 327.9)	-8.61	7 (-4.1; 18.1)	11.3 (5.2; 17.3)	-37.85
**2020 TOTAL**	**1965**	**1807.5**	**8.72**	**2481**	**2825.14**	**-12.18**	**145**	**124.63**	**16.34**
Jan 2021	197 (161.5; 232.5)	185.8 (154.7; 216.9)	6.02	236 (177.7; 294.3)	263.9 (210.2; 317.5)	-10.56	20 (10.0; 30.0)	13.4 (7.3; 19.5)	49.17
Feb 2021	171 (135.4; 206.6)	176.6 (145.2; 207.9)	-3.15	261 (202.5; 319.5)	244.4 (190.5; 298.2)	6.81	15 (5.0; 25.0)	12.4 (6.3; 18.5)	20.90
Mar 2021	241 (205.3 276.7)	195.5 (161.7; 229.3)	23.28	289 (230.4; 347.6)	316.0 (259.6; 372.4)	-8.55	49 (38.9; 59.1)	15.4 (8.6; 22.1)	218.39
Apr 2021	206 (170.2; 241.8)	191.8 (157.8; 225.9)	7.39	241 (182.2; 299.8)	313.4 (256.7; 370.0)	-23.09	55 (44.8; 65.2)	11.4 (4.6; 18.2)	382.89
May 2021	205 (169.1; 240.9)	188.5 (154.2; 222.8)	8.76	259 (200.1; 317.9)	282.7 (225.8; 339.6)	-8.38	35 (24.8; 45.2)	16.4 (9.5; 23.2)	113.55
Jun 2021	200 (164.1; 235.9)	168.2 (133.6; 202.7)	18.93	252 (192.9; 311.1)	261.4 (204.2; 318.5)	-3.58	35 (24.7; 45.3)	17.1 (10.1; 23.9)	105.20
Jul 2021	231 (195.0; 267.0)	157.2 (122.3; 192.0)	46.98	292 (232.8; 351.2)	287.0 (229.6; 344.5)	1.73	24 (13.6; 34.4)	13.1 (6.1; 20.0)	83.82
Aug 2021	205 (168.9; 241.1)	179.2 (144.1; 214.2)	14.42	225 (165.6; 284.4)	275.4 (217.7; 333.1)	-18.29	21 (10.6; 31.4)	9.4 (2.4; 16.4)	123.65
Sep 2021	192 (155.8; 228.2)	157.2 (121.8; 192.5)	22.17	273 (213.5; 332.5)	266.0 (208.1; 324.0)	2.62	9 (-1.5; 19.5)	11.7 (4.7;18.8)	-23.23
Oct 2021	199 (162.7; 235.3)	180.8 (145.2; 216.4)	10.05	258 (198.3; 317.7)	273.0 (214.8; 331.3)	-5.50	13 (2.4; 23.6)	12.1 (5.0; 19.1)	7.83
Nov 2021	182 (145.6; 218.4)	165.8 (130.0; 201.7)	9.75	286 (226.1; 345.9)	261.0 (202.5; 319.5)	9.57	8 (-2.6; 18.6)	11.1 (3.9; 18.2)	-27.64
Dec 2021	185 (148.5; 221.5)	171.2 (135.0; 207.3)	8.08	286 (226.0; 346.0)	273.0 (214.2; 331.8)	4.75	11 (0.3; 21.7)	11.7 (4.6; 18.9)	-6.17
**2021 TOTAL**	**2414**	**2117.69**	**13.99**	**3158**	**3317.15**	**-4.80**	**295**	**155.05**	**90.27**
**TOTAL COVID PERIOD**	**4379**	**3925.14**	**11.56**	**5639**	**6142.29**	**-8.19**	**440**	**279.68**	**57.32**

Table S3. Observed and expected values of maternal mortality ratio (MMR) and under-5 mortality rate (U5MR) in 2020 and 2021 by region in Brazil.

	2020 Observed	2021 Observed	2020 Expected (95%CI)	2021 Expected (95%CI)
**MMR**				
**Brazil**	74.7	110.1	59.5 (54.8; 64.2)	59.0 (53.6; 64.4)
North	98.9	140.9	86.4 (76.6; 96.2)	87.3 (76.0; 98.5)
Northeast	91.8	106.9	66.3 (59.8; 72.7)	64.6 (57.2; 72.1)
Southeast	65.1	100.5	56.9 (50.4; 63.5)	57.6 (50.1; 65.1)
South	45.6	105.2	32.8 (23.9; 41.8)	30.6 (20.4; 40.9)
Central-West	77.0	128.8	57.8 (50.6; 65.1)	56.6 (48.3; 64.9)
**U5MR**				
**Brazil**	14.0	13.8	14.6 (14.0; 15.2)	14.3 (13.6; 15.0)
North	18.9	17.5	18.9 (17.8; 20.0)	18.4 (17.1; 19.6)
Northeast	16.3	15.0	16.6 (15.6; 17.6)	16.2 (15.0; 17.3)
Southeast	12.2	12.5	13.1 (12.7; 13.5)	12.9 (12.4; 13.4)
South	10.4	11.0	11.1 (10.8; 11.5)	10.9 (10.5; 11.4)
Central-West	14.0	13.8	15.0 (14.1; 15.9)	14.6 (13.6; 15.6)

CI - confidence interval
